# Osteopathic Student Matriculation Trends in Surgical Subspecialties (2016-2024): A Systematic Review

**DOI:** 10.1177/23821205261443551

**Published:** 2026-04-25

**Authors:** Bridgette Kielhack, Selena K. Cholak, Virgil K. DeMario, Vaishnavi J. Patel, Devki Patel, Kimberly Toumazos, Mark Quiring, Young Son, John Etlinger

**Affiliations:** 1 Department of Clinical Affairs, 483612 University of the Incarnate Word School of Osteopathic Medicine, San Antonio, TX, USA; 2 Department of Population Health, Division of Family Medicine, 377659 The University of Texas at Austin Dell Medical School, Austin, TX, USA; 3 12342 Texas Tech University Health Science Center School of Medicine, Lubbock, TX, USA; 4 Department of Urology, 4530 University of Kentucky, Lexington, KY, USA; 5 Department of Urology, 20869 Jefferson Health in New Jersey, Voorhees Township, NJ, USA

**Keywords:** osteopathic medical students, residency, trends, COMLEX, USMLE, ACGME

## Abstract

**Background:**

There is a paucity of data on trends of osteopathic medical students (OMS) matriculating into surgical specialties since the installation of the single U.S. graduate medical education (GME) accreditation system, which concluded in June 2020. This study examines the impact of major policy changes, particularly the transition of COMLEX Level 1 and USMLE Step 1 to Pass/Fail, on OMS match outcomes, with the goal of guiding current students pursuing surgical careers.

**Methods:**

A systematic literature review was conducted using Rayyan, with bias assessed via the Cochrane risk of bias tool. National Residency Matching Program (NRMP) data from 2016, 2018, 2020, 2022, and 2024 were reviewed to document the number of OMS applicants and post-match statistics. Surgical specialties analyzed included General Surgery, Vascular Surgery, Neurological Surgery, Otolaryngology, and Orthopedic Surgery. NRMP pre-merger data were available for General Surgery, Neurological Surgery, and Orthopedic Surgery; Vascular Surgery and Otolaryngology data were available for 2020 and 2022 only.

**Results:**

COMLEX-USA Level 1/2 and USMLE Step 1/2 scores generally decreased across the ACGME merger, with notable exceptions: Neurological Surgery reported higher COMLEX Level 2-CE scores; Orthopedic Surgery reported higher USMLE Step 2 CK scores; Vascular Surgery reported a higher Step 2 score between 2020 and 2022, though data were limited to a single applicant. Otolaryngology also demonstrated an increase in mean Step 2 scores. General Surgery, Neurological Surgery, and Orthopedic Surgery showed increased mean numbers of abstracts, presentations, and publications, while Vascular Surgery and Otolaryngology showed declines between 2020 and 2022.

**Conclusions:**

The most consistent trend was an increase in research productivity among successful OMS matriculating into General Surgery, Neurological Surgery, and Orthopedic Surgery. While licensing scores generally declined, this may reflect applicant pool expansion post-merger. Ongoing research is needed to evaluate trends beyond 2024, particularly following the implementation of Pass/Fail Step/Level 1.

## Introduction

Historically, the majority of osteopathic medical students (OMS) matriculated into primary care residency programs at increasing rates.^1^ In 2020 and 2023, DO seniors comprised 20.85% and 22.5% of matched applicants in primary care specialties, respectively.^1^ In contrast, from 2018 to 2023, DO seniors only comprised 1.11% to 1.05% of matched applicants into surgical subspecialties, respectively.^1^ The Department of Health and Human Services projects there to be workforce shortages in most surgical subspecialties by 2025, specifically in general surgery, urology, orthopedic surgery, and ophthalmology.^2^ More than 100 rural hospitals closed within the past decade partially due to loss of surgical services.^3^ Consequently, the remaining urban hospitals are becoming burdened with more surgical cases due to the increasing number of patients coming in from neighboring rural and suburban areas. One way to alleviate this burden is to increase the number of osteopathic students matriculating into surgical subspecialties. Interestingly, the 2023 Osteopathic Medical Profession (OMP) Report showed a more than 30% increase in the number of osteopathic physicians in the United States in the past five years.^4^ Prior to the creation of the Accreditation Council for Graduate Medical Education (ACGME) in 2020, osteopathic medical students encountered relatively few challenges as residency surgical specialty spots were equally available to both MD and DO students. However, after the inception of the ACGME/AOA merger, residency positions that were previously exclusive for DO graduates now became equally accessible to both MD and DO students, transforming the applicant pool to a more competitive environment due to the inclusion of MD students.^5^ As a result, residency programs are now considering additional factors in their selection of prospective residents.

Current literature demonstrates that scoring above average on licensing exams is critical for both allopathic and osteopathic students matching into their first-choice residency and into more competitive specialties.^6^ The quantity of research experience, which includes abstracts, presentations, and publications, is important in establishing oneself as a competitive applicant. A study published by Hasley et al indicated that medical students who matched into a highly competitive specialty, neurosurgery, had an average of 5.1 publications and a median of 4.0 publications per resident.^7^ In a study regarding osteopathic students matriculating into orthopedic surgery, performance in rotating at a program directors’ (PD) institution, formality/politeness in interview, performance on ethical questions in interview, and COMLEX/USMLE Step 1/Step 2 scores were the top five residency criteria selection criteria.^8^ This suggests that medical students applying to surgical subspecialty residency programs must display higher-level licensing exam scores along with research authorship. Using this foundation, we aim to provide a thorough examination of the patterns of osteopathic medical students’ matriculation into surgical special programs.

Through an extensive systematic review, our aim is to provide a comprehensive analysis of trends of matriculation of osteopathic medical students into surgical specialty programs. The findings of this research are intended to facilitate a deeper understanding and serve as foundational evidence for the prevailing trends of osteopathic medical students into non-primary care surgical specialty residency programs. The results can be utilized by prospective and current students, medical educators, physicians, and residency directors to advance diversity, equity, and inclusion initiatives of osteopathic medical students into non-primary care specialties within the medical community.

## Methods

This systematic review was conducted in accordance with the PRISMA 2020 guidelines to examine trends in osteopathic medical student matriculation into surgical subspecialties following the ACGME/AOA merger and the transition of USMLE Step 1 and COMLEX Level 1 to Pass/Fail.

### Data Sources and Study Design

Two data sources were used: (1) peer-reviewed literature and (2) publicly available National Residency Matching Program (NRMP) applicant-level data. NRMP data from the 2016, 2018, 2020, 2022, and 2024 Main Residency Match cycles were analyzed to document osteopathic senior applicant characteristics and post-match outcomes. Surgical specialties examined included General Surgery, Vascular Surgery, Neurological Surgery, Otolaryngology, and Orthopedic Surgery. Pre-merger NRMP data were available for General Surgery, Neurological Surgery, and Orthopedic Surgery, whereas Vascular Surgery and Otolaryngology data were available for 2020 and 2022 only.

Because all data were obtained from publicly available sources, Institutional Review Board approval and informed consent were not required, and no external funding was received.

### Search Strategy

A comprehensive literature search was conducted in PubMed/MEDLINE, Embase, Web of Science, and Scopus to identify studies evaluating osteopathic medical students or physicians in relation to surgical specialties, internship and residency training, and residency match outcomes. Searches included articles published between January 1, 2012 and December 31, 2022. The final search was completed on December 31, 2022.

In PubMed, Medical Subject Headings (MeSH) and free-text keywords were combined. The full strategy was: (“specialties”[MeSH Terms] OR “surgical”[MeSH Terms] OR “specialties”[All Fields] OR “surgical”[All Fields]) AND (“internship”[MeSH Terms] OR “residency”[MeSH Terms] OR “internship”[All Fields] OR “residency”[All Fields] OR “residency program”[All Fields]) AND (“osteopathic students”[MeSH Terms] OR “osteopathic medicine”[MeSH Terms] OR “osteopathic physician”[MeSH Terms] OR “osteopathic”[All Fields] OR “DO”[All Fields] OR “osteopathic medical student”[All Fields]), limited to English-language publications from 2012 to 2022.

In Embase, exploded Emtree terms were used: (“osteopathic students”/exp OR “osteopathic medicine”/exp OR “osteopathic physician”/exp OR “osteopathic medical student”/exp OR “osteopathic”/exp OR “DO”/exp) AND (“residency match”/exp OR “match process”/exp OR “residency matching”/exp OR “residency placement”/exp) AND (“surgical specialties”/exp OR “specialties”/exp OR “surgical”/exp OR “internship”/exp OR “residency”/exp).

**Figure 1.**
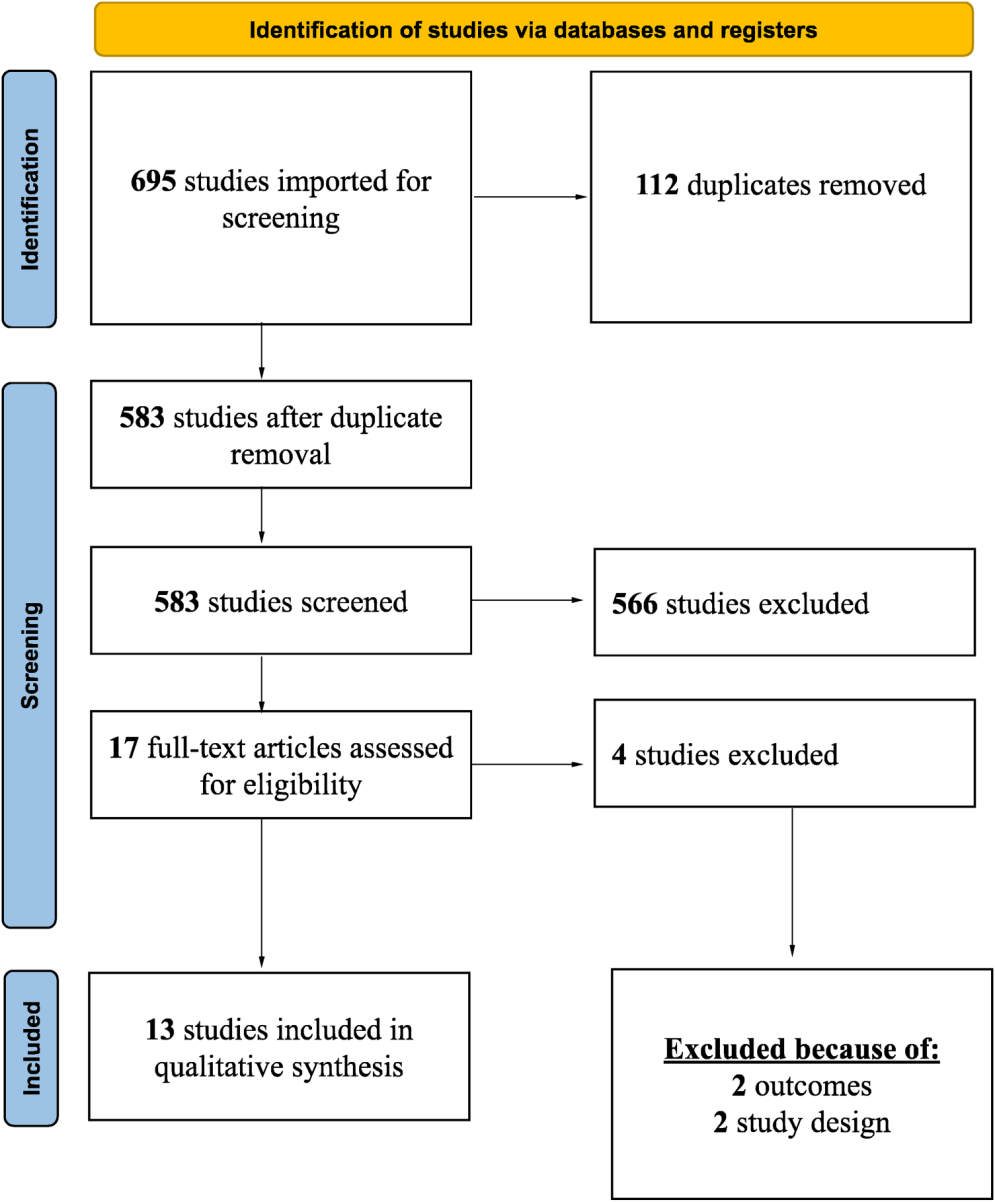
PRISMA flow chart.

In Web of Science and Scopus, analogous keyword-based strategies were used, with Scopus restricted to publications after 2012. The study selection process is summarized in the PRISMA flow diagram (Figure 1).

### Eligibility Criteria

Studies were eligible for inclusion if they were peer-reviewed, published in English, and reported outcomes related to osteopathic medical students or physicians in surgical specialties, including residency match trends, licensing examination performance, or research productivity. Studies were excluded if they were editorials, commentaries, non-English publications, not specific to osteopathic applicants, or lacked relevant outcome data.

### Study Selection

All records were imported into Rayyan for de-duplication and screening. Two reviewers independently screened titles and abstracts, followed by full-text review for eligibility. Disagreements were resolved by consensus. A total of 695 records were identified, 17 underwent full-text review, and 13 were included in the qualitative synthesis.

### Data Extraction

Two reviewers independently extracted data from each included study. Extracted variables included study year, study design, surgical specialty, applicant characteristics, board score reporting, research productivity, and match outcomes. When data were unclear, original publications were reviewed for clarification.

### Outcomes and Variables

The primary outcomes of interest were licensing examination performance (COMLEX and USMLE), research productivity (abstracts, presentations, publications, and research experiences), and residency match outcomes. Additional variables included surgical specialty, match cycle year, and study design. All reported values compatible with these outcome domains were collected.

### Risk of Bias Assessment

**Figure 2.**
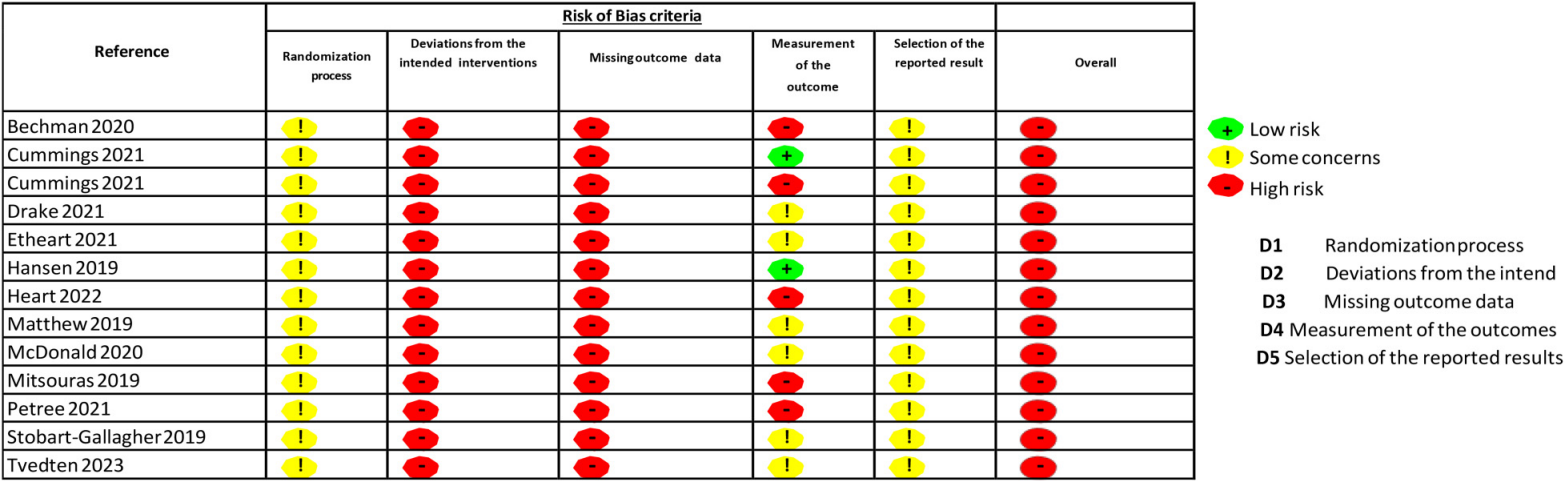
Risk of bias criteria.

Risk of bias was assessed using the Cochrane Risk of Bias Tool. Two reviewers independently evaluated each included study, with disagreements resolved by consensus. Results are summarized in Figure 2.

### Data Synthesis and Statistical Analysis

This study was not registered with PROSPERO or INPLASY because it analyzed publicly available NRMP data and retrospective literature to describe educational and workforce trends. NRMP and literature-derived data were summarized using descriptive statistics, including means, counts, and percentages. Data were grouped by surgical specialty and match cycle (pre-merger vs post-merger) to evaluate trends following the ACGME/AOA merger and the transition to Pass/Fail licensing. No meta-analysis, heterogeneity assessment, sensitivity analysis, or certainty grading was performed due to study design heterogeneity and the descriptive nature of available data.

## Results

### Literature Review Data

Seven of the nine authors (78%) considered the United States Medical Licensing Exam (USMLE) Step 1 “significantly important” for matching into residency subspecialties (Table 1).^6,8-14^ Heard 2022 reported in their study that residency programs recommended DO applicants to take the USMLE Step 1 exam in order to be considered “equal” to their allopathic counterparts.^11^ Moreover, 67% of studies stated that taking the USMLE Step 2 CK exam may increase competitiveness to match residency subspecialties (Table 1).^6,8,10-14^ Regarding numerical values to target, Mitsouras 2019 reported that 53.9% of graduates matched into their first choice residency program had a mean USMLE Step 2 CK score of 228.1 points.^6^ Stobart-Gallagher 2019 recommended test-takers to score above 240 points to match into emergency medicine, following the single accreditation system in 2020.^12^

**Table 1.** Main Characteristics of Studies Included in the Systematic Review.

First Author	Title	Year			Type of Study			Sample Characteristics		
Beckman	Characteristics of ACGME Residency Programs That Select Osteopathic Medical Graduates	2020			N/A			Data were analyzed from AMA's Fellowship and Residency Electronic Interactive Database Access (FREIDA) to determine characteristics of programs that reported DO residents		
Drake	Characteristics of Matriculants to Thoracic Surgery Residency Training Programs	2021			Retrospective Study			Matriculation status data and match statistics for MDs and DOs were gathered from ERAS from 2014 to 2017		
Hansen	The Osteopathic Applicant	2019			N/A			Recommendations for osteopathic medical students interested in applying to Emergency Medicine residency programs		
Heard	Identifying Attitudes Toward and Acceptance of Osteopathic Graduates in Surgical Residency Programs in the Era of Single Accreditation: Results of the American Collect of Osteopathic Surgeons Medical Student Section Questionnaire of Program Directors	2022			Questionnaire			Surgical specialties included: Orthopedic Surgery, ENT, General Surgery, Urology, NSGY, Integrated Vascular Surgery, Integrated Thoracic Surgery, and Integrated Plastic Surgery. 19% were former AOA-accredited GME programs. Almost half of the programs indicated they are not training DOs currently		
McDonald	Osteopathic Orthopedic Residency Selection Criteria: Program Directors’ Survey and Analysis	2020			Questionnaire survey			Twenty-five-question survey was completed by program directors of osteopathic orthopedic programs in 2017		
Mitsouras	Student Academic Performance Factors Affecting Matriculating into First-Choice Residency and Competitive Specialties	2019			Retrospective study			Pre-medical data and match statistics were analyzed for successful matriculation into first choice residency programs and competitive specialties		
Stobart	Emergency Medicine Residency Directors: Osteopathic Applicants	2019			N/A			A sub-group of the Advising Students Committee of Emergency Medicine (ASC-EM) proposes recommendations for osteopathic medical students after the shift to the Single Accrediting System in 2020		
Tvedten	Attitudes Toward Allopathic and Osteopathic Candidates in the Dermatologic Residency Application Process	2023			Retrospective Study; Online Survey-based study			Surveyed MD/DO dermatology residents and faculty and their attitudes, beliefs, and experiences in successful matriculation into dermatology programs from August 2021 to September 2021		

First Author	Title	COMLEX Level 1 Score	COMLEX Level 2 Score	USMLE Step 1 Score	USMLE Step 2 Score	Taking Only COMLEX	Taking USMLE & COMLEX
Beckman	Characteristics of ACGME Residency Programs That Select Osteopathic Medical Graduates			Higher USMLE Step scores were associated with residency programs that reported lower number of DO residents (programs were general surgery and surgery subspecialty programs)			
Drake	Characteristics of Matriculants to Thoracic Surgery Residency Training Programs						
Hansen	The Osteopathic Applicant			Recommended students to take USMLE Step 1	Recommended students to take USMLE 2 and improvement in score from 1 to 2 can increase competitiveness	Yes	Yes
Heard	Identifying Attitudes Toward and Acceptance of Osteopathic Graduates in Surgical Residency Programs in the Era of Single Accreditation: Results of the American Collect of Osteopathic Surgeons Medical Student Section Questionnaire of Program Directors			Residency programs recommended taking USMLE Step 1 to be considered “equal” to their MD counterparts	Residency programs recommended taking to be considered “qual” to their MD counterparts	Yes	
McDonald	Osteopathic Orthopedic Residency Selection Criteria: Program Directors’ Survey and Analysis			PDs recommended taking USMLE Step 1	PDs recommend taking USMLE Step 2		PDs recommend taking USMLE and STEP exams
Mitsouras	Student Academic Performance Factors Affecting Matriculating into First-Choice Residency and Competitive Specialties	53.9% of graduates matched into their first-choice residency program with a mean score of 514.2 points	53.9% of graduates matched into their first-choice residency program with a mean score of 522.5 points	53.9% of graduates matched into their first-choice residency program with a mean score of 213.4 points	53.9% of graduates matched into their first-choice residency program with a mean score of 228.1 points		
Stobart-Gallagher	Emergency Medicine Residency Directors: Osteopathic Applicants			Recommended scoring greater than 235 points	Recommend scoring above 240 points		Recommend taking USMLE 1 and 2 in addition to COMLEX Level 1 and 2
Tvedten	Attitudes Toward Allopathic and Osteopathic Candidates in the Dermatologic Residency Application Process	Significantly important	Significantly important	Significantly important	Significantly important		Taking both exams are important as more than half the allopathic faculty reported they had “no comfort” in converting COMLEX scores

First Author	Title	AOA Membership	Number of Publications	Number of Research Projects	Clerkship Grades	Surgery Residency Affiliated with Surgical Subspecialty	Completing Audition Rotations	Home Program	Mentorship	STEP/COMLEX 1 Becoming Pass/Fail
Beckman	Characteristics of ACGME Residency Programs That Select Osteopathic Medical Graduates									The data for this article were received by 2/2019 before STEP/COMLEX was P/F which recorded that higher board scores were associated with programs reporting lower number of DO students compared to specialty
Drake	Characteristics of Matriculants to Thoracic Surgery Residency Training Programs	92% of matriculants were AOA members, but this value only applies to US MD school graduates	Median was three publications per applicant for matriculated students			85% of matriculants attended a TS residency-affiliated with GS residency		Home programs affiliation is not statistically significant with a P value > .999		
Hansen	The Osteopathic Applicant						Recommend rotating at institution you want to apply to	Notes that some institutions require students to do EM rotation at home institution	Recommend students to find mentors that can guide them through the application process	
Heard	Identifying Attitudes Toward and Acceptance of Osteopathic Graduates in Surgical Residency Programs in the Era of Single Accreditation: Results of the American Collect of Osteopathic Surgeons Medical Student Section Questionnaire of Program Directors			Residency programs recommend increasing volume of research projects			Residency programs recommend to complete audition rotations (sub-internships)		Residency program recommend DO students to seek out mentorship early on in their medical school career	
McDonald	Osteopathic Orthopedic Residency Selection Criteria: Program Directors’ Survey and Analysis		Overall, published research experience was ranked 9 out of 17 criteria of importance	Overall, research experience with no publication was ranked 10 out of 17 criteria of importance			PDs recommend students to rotate at PD's institution			
Mitsouras	Student Academic Performance Factors Affecting Matriculating into First-Choice Residency and Competitive Specialties									
Stobart-Gallagher	Emergency Medicine Residency Directors: Osteopathic Applicants						Recommend completing at least two EM rotations at academic institutions for two SLOE recommendation letters			
Tvedten	Attitudes Toward Allopathic and Osteopathic Candidates in the Dermatologic Residency Application Process			Significantly important	Significantly important					

### Match Statistics Trends from NRMP Data upon STEP/LEVEL 1 Becoming Pass/Fail

DO Seniors that matriculated into General Surgery, Orthopedic Surgery, and Otolaryngology had a higher research volume compared to matriculants from previous years. General surgery residents had a high mean number of abstracts, presentations, and publications (6.9) post-merger compared to pre-merger in 2022 at 4.6. Similarly, the mean number of research experiences was higher for general surgery residents in 2024 compared to 2022 at 3.5 and 3, respectively (Table 2f). Orthopedic surgery residents followed this same trend. Post-merger, the mean number of abstracts, presentations, and publications post-merger (2024) for orthopedic surgery was 11.2, compared to 7 pre-merger (2022). The mean number of research experiences post-merger was 5.1, compared to 4 pre-merger (2022) Table 2h). Otolaryngology residents had a mean number of abstracts, presentations, and publications of 11.3 post-merger (2024), which was higher than the pre-merger at 8.5 (2022). Interestingly, the mean number of research experiences for otolaryngology post-merger was 4.5, decreased from the pre-merger of 11.2 (2022) (Table 2j).

**Table 2.** Board Scores and Research.

	Mean COMLEX-USA Level 1 Score	Mean COMLEX-USA Level 2 CE Score	Mean USMLE Step 1 Score	Mean USMLE STEP 2 CK Score
(a) General Surgery Board Scores						
Year 2016	609	618		
Year 2018	613	666	238	248
Year 2020	585	617	231	243
Year 2022	590	626	232	246

	Mean COMLEX-USA	Mean COMLEX-USA	Mean USMLE	Mean USMLE
	Level 1 Score	Level 2 CE Score	Step 1 Score	STEP 2 CK Score
(b) Neurological Surgery Board Scores				
Year 2018	621	529	252	252
Year 2020	568	584	252	231
Year 2022	639	643	246	253

	Mean COMLEX-USA	Mean COMLEX-USA	Mean USMLE	Mean USMLE
	Level 1 Score	Level 2 CE Score	Step 1 Score	STEP 2 CK Score
(c) Orthopedic Surgery Board Scores				
Year 2018	680	680	252	249
Year 2020	656	677	242	250
Year 2022	639	675	243	250

	Mean COMLEX-USA	Mean COMLEX-USA	Mean USMLE	Mean USMLE
	Level 1 Score	Level 2 CE Score	Step 1 Score	STEP 2 CK Score
(d) Vascular Surgery Board Scores.				
Year 2020	629	627	243	250
Year 2022	573	601	246	241

	Mean COMLEX-USA	Mean COMLEX-USA	Mean USMLE	Mean USMLE
	Level 1 Score	Level 2 CE Score	Step 1 Score	STEP 2 CK Score
(e) Otolaryngology Board Scores				
Year 2020	660	697	240	252
Year 2022	655	681	246	249

	Mean Number of Research Experiences		Mean Number of Abstracts, Presentations, and Publications	
(f) General Surgery Research				
Year 2016	2		2.7	
Year 2018	2		3.2	
Year 2020	2.7		3.6	
Year 2022	3		4.6	

	Mean Number of Research Experiences		Mean Number of Abstracts, Presentations, and Publications	
(g) Neurological Surgery Research				
Year 2018	7.3		21.3	
Year 2020	3		11.5	
Year 2022	7.8		32.6	

	Mean Number of Research Experiences		Mean Number of Abstracts, Presentations, and Publications	
(h) Orthopedic Surgery Research				
Year 2018	2.7		4	
Year 2020	3.3		6.7	
Year 2022	4		7	

	Mean Number of Research Experiences		Mean Number of Abstracts, Presentations, and Publications	
(i) Vascular Surgery Research				
Year 2020	3.8		21.3	
Year 2022	5		16	

	Mean Number of Research Experiences		Mean Number of Abstracts, Presentations, and Publications	
(j) Otolaryngology Research				
Year 2020	4.4		6.1	
Year 2022	11.2		8.5	

DO seniors that matriculated into neurological surgery and vascular surgery residency programs had a lower research volume compared to matriculants from previous years. Vascular surgery residents had a mean number of abstracts, presentations, and publications of 9 post-merger (2024), decreased from 15 in the pre-merger (2022). Similarly, the mean number of research experiences was 2.8 post-merger (2024), decreased from pre-merger at 5 (2022) (Table 2i). Incoming neurological surgery residents also displayed lower research volume. The mean number of abstracts, presentations, and publications post-merger was 23 (2024), lower than incoming neurological surgery residents had pre-merger at 32.6 (2022). Similarly, the mean number of research experiences post-merger for neurological surgery residents was 4 post-merger (2024), decreased from 7.8 pre-merger (2022) (Table 2g).

### Match Statistic Trends from NRMP Pre-ACGME Merger (2016/2018) and Post-ACGME Merger (2020/2022) Were Analyzed

NRMP match statistics across the ACGME merger (2016-2018 to 2020-2022) reported a decrease in scores for the COMLEX Level 1 for General surgery, Neurosurgery, and Orthopedic surgery, Vascular surgery, and Otolaryngology (Table 2a-e).^15-18^ There was a decrease in COMLEX Level 2-CE scores post-merger for General surgery, Orthopedic surgery, Vascular surgery, and Otolaryngology; while there was an increase post-merger for Neurological surgery (Table 2a, c, d, e, and i).^15-18^ There was a decrease in USMLE Step 1 exam scores for General surgery, Neurological surgery, and Orthopedic surgery (2.a, 2.c, and 2.e).^15-18^ There was an increase in USMLE Step 1 exam scores from 2020 to 2022 for Vascular surgery and Otolaryngology (Table 2b and d).^15-18^ There was a decrease in USMLE Step 2 CK exam scores for General surgery, Neurological surgery, Vascular surgery, and Otolaryngology (Table 2a-d).^15-18^ There was an increase in USMLE Step 2 CK score for Orthopedic surgery across the ACGME merger (Table 2d).^15-18^ Finally, the mean number of abstracts, presentations, and publications increased across the ACGME merger for General surgery, Neurological surgery, and Orthopedic surgery (Table 2f-h).^15-18^ From 2020 to 2022, the mean number of abstracts, presentations, and publications decreased for vascular surgery and otolaryngology (Table 2g and h).^15-18^

### ACGME Merger Effect on Matriculation (NRMP DATA)

**Figure 3.**
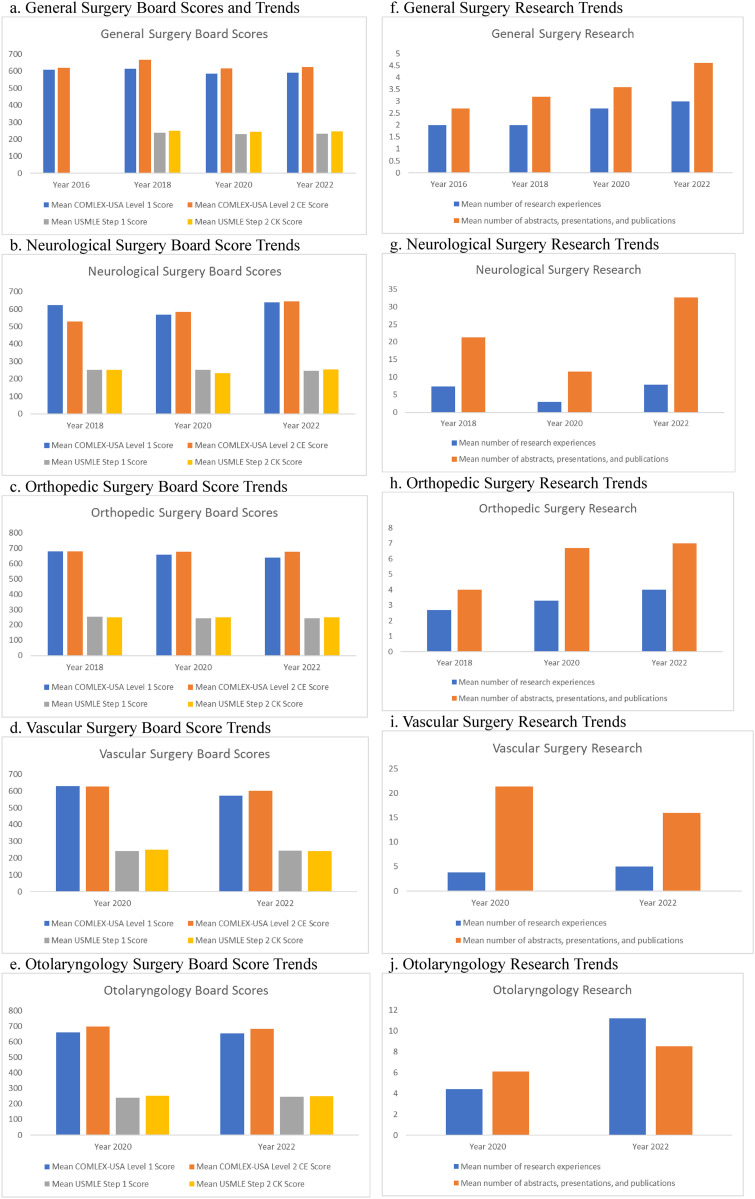
Board score and research trends.

Following the ACGME merger, the mean COMLEX-USA Level 1 score decreased for General surgery, Neurological surgery, Orthopedic surgery, Vascular surgery, and Otolaryngology (Table 2a-e).^15-18^ From 2016 to 2018, the mean score for General surgery was 611 points for successful matriculants (Figure 3a).^15-18^ From 2020 to 2022, the mean score for General surgery was 587.5 points (Figure 3a).^15-18^ This is almost a 4% decrease from the pre-ACGME merger NRMP data. The stark increase in number of students applying to surgical specialties across the merger may account for the variability in scores. In 2016 and 2018, 100 and 121 DO applicants participated in the ACGME match, respectively.^15-18^ Whereas in 2020 and 2022, there were 254 and 264 DO applicants post-ACGME merger.^15-18^ In 2018, the mean score for Neurological surgery was 621 points with three successful matriculants (Figure 3b).^15-18^ In 2020, two DO applicants matched with a mean score of 568 points (Figure 3b).^15-18^ In 2022, nine matriculants had a mean score of 639 points (Figure 3b).^15-18^ Therefore, while there was a decrease in mean scores, this does not account for the fact that there was an increased number of neurosurgical applicants. For Orthopedic surgery, the mean COMLEX-Level 1 score was 680 points with four matriculants (Figure 3c).^15-18^ From 2020 to 2022, the mean scores for Vascular surgery were 629 and 573 points, respectively (Figure 3d).^15-18^ The mean scores for Otolaryngology also decreased from 660 points in 2020 to 655 points in 2022 (Figure 3e).^15-18^

Neurological surgery matriculants had a 16% increase in COMLEX-LEVEL 2 exam scores across the ACGME merger while mean scores decreased for General surgery, Orthopedic surgery, Vascular surgery, and Otolaryngology (Figure 3a-e).^15-18^ Pre-merger, Neurological surgery matriculants had a mean score of 529 points while post-merger, the mean score was 613.5 points which is a 16% increase (Figure 3b).^15-18^ For General surgery, the mean score pre-merger was 642 points while the mean score post-merger was 621.5 points which was a 3% decrease (Figure 3a).^15-18^ For Orthopedic surgery, the pre-merger score was 680 points for successful matriculants, and the post-merger mean score was 676 points which was a 0.6% decrease (Figure 3c).^15-18^ For Vascular surgery matriculants, there was a 4% decrease in scores from 627 in 2020 to 601 in 2022 (Figure 3d).^15--18^ ENT matriculants also had a 2.3% decrease from 697 in 2020 to 681 in 2022 (Figure 3e).^15-18^

Regarding USMLE Step 1 exam scores, General surgery, Neurological surgery, and Orthopedic surgery, matriculants reported a decrease across the ACGME merger (Figure 3a-c).^15-18^ For General surgery, there was a 3% decrease in scores from 238 points pre-merger to 231.5 points post-merger (Figure 3a).^15-18^

Neurological surgery matriculants reported a 1% decrease in mean USMLE Step 1 exam scores from 252 points pre-merger to 249 points post-merger (Figure 3b).^15-18^ Orthopedic surgery matriculants reported a 3.7% decrease in USMLE Step 1 exam scores across the ACGME merger from 252 points pre-merger to 242.5 points post-merger (Figure 3c).^15-18^ In contrast, Vascular surgery and Otolaryngology matriculants reported an increase in board scores from 2020 to 2022. In 2020, the mean USMLE Step 1 score for vascular surgery and Otolaryngology were 243 and 240 points, respectively (Figure 3d and e).^15-18^ The mean score in 2022 was 246 points for both surgical subspecialties (Figure 3d and e).^15-18^

General surgery, Neurological surgery, Vascular surgery, and Otolaryngology reported a decrease in USMLE Step 2 CK scores from 2018 to 2022 while Orthopedic surgery matriculants reported an increase in USMLE Step 2 CK scores (Figure 3a-e).^15-18^ Orthopedic surgery matriculants reported a 0.4% increase in board scores from 249 points in 2018 to 250 points post-merger (Figure 3c).^15-18^ General surgery saw a 1.4% decrease from 248 pre-merger to 244.5 points post-merger (Figure 3a).^15-18^ Neurological surgery matriculants reported a 7.5% decrease from 252 points pre-merger to 242 points post-merger (Figure 3b).^15-18^ Though, this might be a skewed result since only two matriculants matched in 2020 with an average score of 231 points while nine students matched in 2022 with a mean USMLE Step 2 CK score of 253 points (Figure 3b).^15-18^ In 2020, Vascular surgery matriculants reported a mean score of 250 points compared to a mean score of 241 points in 2022 (Figure 3d).^15-18^ In 2020, Otolaryngology matriculants reported a mean score of 252 points in 2020 compared to a mean score of 249 points in 2022 (Figure 3e).^15-18^

Across the ACGME merger, the mean number of abstracts, presentations, and publications increased for those that matched into General surgery, Neurological surgery, and Orthopedic surgery (Figure 3f-h).^15-18^ Vascular surgery and otolaryngology matriculants reported a decrease in the mean number of abstracts, presentations, and publications from 2020 to 2022 (Figure 3i and j).^15-18^ General surgery matriculants reported a 39% increase across the ACGME merger with a mean number of 4.1 research experiences post-merger (Figure 3f).^15-18^ Neurological surgery matriculants reported a 3.5% increase across the ACGME merger with a mean number of 22 research experiences post-merger (Figure 3g).^15-18^ Orthopedic surgery matriculants reported a 71.3% increase across the ACGME merger with a mean number of 6.85 research experiences post-merger (Figure 3h).^15-18^ Finally, while Vascular surgery saw a decline in the number of abstracts, presentations and publications from 2020 to 2022, this could be due to the fact that there was only one person who matched in 2022 (Figure 3i).^15-18^ In 2020, Vascular surgery matriculants reported a mean number of 21.3 abstracts, presentations and publications while in 2022, there was a mean number of 16 (Figure 3g).^15-18^ Otolaryngology matriculants reported a mean number of 11.2 research experiences in 2020 and 8.5 research experiences in 2022 (Figure 3j).^15-18^

## Discussion

### Research Volume

Following the ACGME/AOA merger, DO seniors who matched into General surgery, Neurological surgery, and Orthopedic surgery obtained a higher number of abstracts, presentations, and publications (Figure 3f-h).^15-18^ Based on match statistics data from 2016 and 2018, Matthews et al posits that for allopathic students, there is a statistically significant interaction between match success and the number of research accomplishments.^19^ In contrast, there was no statistically significant interaction between being a DO student and matching success.^19^ Notably, this research pertains to all specialties and is not specific for surgical subspecialties.

The merger provided more surgical residency positions for DO students. In 2018, 268 AOA positions were explicitly available for DO seniors in the following surgical specialties: General surgery, Neurological surgery, Orthopedic surgery, and Otolaryngology.^1^ By 2023, there were a total of 3535 positions for DO and MD seniors made possible with the ACGME-AOA merger.^1^ But even with increased availability, MD seniors occupied the majority of these positions. For example, from 2020 to 2023, match rates were higher for MDs than DOs in neurosurgery, thoracic surgery, plastic surgery, general surgery, vascular surgery, ENT, and orthopedics.^1^ Although the ACGME/AOA merger ultimately increased the total number of residency positions available, the proportion of DO students matching into competitive surgical subspecialties decreased due to a larger applicant pool and greater competition from MD graduates, highlighting that increased positions do not necessarily translate to higher relative match rates for DO students.

Because research volume serves a significant role in match success for allopathic seniors, it is unsurprising that there is an increase in research volume for DO seniors across the ACGME merger who are matching into historically MD-dominant surgical subspecialties.^1^ Therefore, it is highly advised that DO seniors who are applying to surgical specialties obtain research experiences in order to be considered competitive. Future research should be done to determine if there are statistically significant interactions between being a DO student, research volume, and matching into surgical subspecialties across the ACGME merger, especially given the growing number of positions in surgical subspecialties.

### Research Volume After COMLEX and STEP Became Pass/Fail

The change to COMLEX Level 1 and USMLE Step 1 Pass/Fail system led to notable differences in how medical students demonstrate their competitiveness to residency subspecialties. General surgery, Orthopedic surgery, and Otolaryngology saw an increase in research volume in applicants.^20^ Previously, COMLEX Level 1 and USMLE Step 1 scores carried significant weight in an applicant's level of competitiveness. With the advent of the Pass/Fail system, students must highlight other characteristics that differentiate themselves from their peers. This change is reflected in the higher research volume among DO Seniors who matriculated into General Surgery, Orthopedic Surgery, and Otolaryngology specialties compared to matriculants from previous years. This shift further suggests that applicants continue to place great emphasis on academic and scholarly achievements to strengthen their residency applications.

### Board Scores

We hypothesized that COMLEX Level 2-CE and USMLE Step 2 CK scores would increase post-merger due to increased competitiveness with their allopathic counterparts. However, due to the bigger pool of applicants, it may be unsurprising that the standardized scores were only a few points lower post-merger for General surgery, Neurological surgery, and Orthopedic surgery with some fluctuations (Figure 3f-h).^15-18^ The major takeaway is there seems to be an ideal range that DO students should prepare for when sitting for their licensing examinations as seen in Figure 3.^15-18^ Therefore, while there is technically a minimal drop in points across the merger, there seems to be a rough estimate for “good numbers to hit” for a specific surgical specialty. Therefore, DO students should aim for these numbers when sitting for their licensing examinations.

### Implications for Advising and Education

These findings have practical implications for advising osteopathic medical students interested in surgical subspecialties. Early exposure and advising during the first and second years of medical school may be particularly important in the post-Pass/Fail era, as students may need additional time to pursue research opportunities, build scholarly portfolios, and identify mentors within competitive surgical fields. Longitudinal mentorship, including guidance on research engagement, audition rotations, and application strategy, may help osteopathic students remain competitive following the transition to Pass/Fail licensing examinations. At an institutional level, targeted support structures such as protected research time, access to surgical faculty mentors, and transparent advising resources may further facilitate osteopathic student matriculation into surgical subspecialties.

### Limitations of This Study

Limitations were that the literature search included language restrictions to only English articles. Additionally, various methodologies were utilized in studies meeting criteria and so the relationship of sample size and efficacy was not able to be successfully analyzed. Additionally, these are self-reported data. Several surgical subspecialties included in this analysis were characterized by small numbers of osteopathic matriculants in individual match cycles, particularly Vascular Surgery and Otolaryngology. In these specialties, year-to-year fluctuations in applicant counts may disproportionately influence mean values for licensing examination scores and research productivity, thereby limiting external validity. As a result, observed post-merger trends for these subspecialties should be interpreted cautiously and viewed as descriptive rather than representative of broader longitudinal patterns. In addition, this study did not include direct degree-matched comparisons between DO and MD applicants within the same surgical subspecialties. The absence of direct DO-MD comparative analyses limits contextual interpretation of relative competitiveness, board score expectations, and research productivity across degree pathways. In addition, while our study highlights research productivity as a factor in residency selection, the data available do not allow us to examine whether greater research output correlates with higher licensing exam scores, stronger residency evaluations, or long-term academic and scholarly success; an important area for future investigation.

## Conclusion

Successful DO matriculants should possess key attributes, such as strong licensing examination scores and research experience. From 2018 to 2020, DOs matching into surgical specialties decreased by 3%, yet the osteopathic profession remains vital in meeting hospitals’ surgical demands. Advocacy and matriculation trend analysis can support DO applicants in securing surgical careers. Research volume increased across General Surgery, Neurological Surgery, and Orthopedic Surgery, aligning with trends among allopathic applicants. While licensing scores generally declined, this may reflect the larger applicant pool following the ACGME/AOA merger. Program directors emphasize the importance of audition rotation performance for surgical residency success. These findings underscore the importance of early, structured advising and longitudinal mentorship for osteopathic students pursuing surgical subspecialties, particularly in the post-Pass/Fail era. Institutional investment in research infrastructure and specialty-specific advising may further support osteopathic students navigating an increasingly competitive surgical match environment. Future studies should explore trends beyond 2022, considering the ACGME merger and the shift to Pass/Fail Level/Step 1 exams.

## Supplemental Material

sj-docx-1-mde-10.1177_23821205261443551 - Supplemental material for Osteopathic Student Matriculation Trends in Surgical Subspecialties (2016-2024): A Systematic Review Supplemental material, sj-docx-1-mde-10.1177_23821205261443551 for Osteopathic Student Matriculation Trends in Surgical Subspecialties (2016-2024): A Systematic Review by Bridgette Kielhack, Selena K. Cholak, Virgil K. DeMario, Vaishnavi J. Patel, Devki Patel, Kimberly Toumazos, Mark Quiring, Young Son and John Etlinger in Journal of Medical Education and Curricular Development

## References

[1] Brazdzionis J Savla P Oppenheim R , et al. Comparison of osteopathic and allopathic candidates matching into selected surgical subspecialties. Cureus. 2023;15(6):e40566. doi:10.7759/cureus.40566 PMC10351620 37465803

[2] Haskins J . Desperately Seeking Surgeons. Association of American Medical Colleges; April 26, 2019. Accessed December 2023. https://www.aamc.org/news/desperately-seeking-surgeons

[3] McCarthy S Moore D Smedley WA , et al. Impact of rural hospital closures on health care access. J Surg Res. 2021;258:170-178. doi:10.1016/j.jss.2020.08.055 33011448

[4] American Osteopathic Association. Osteopathic Medical Profession Report. Accessed December 2023. https://osteopathic.org/wp-content/uploads/2023-OMP-Report.pdf. Published 2023.

[5] Cummings M . The impact of the ACGME/AOA single accreditation system on osteopathic surgical specialties, residents, and DO students. J Surg Educ. 2021;78(5):1469-1475. doi:10.1016/j.jsurg.2021.02.006 33766543

[6] Mitsouras K Dong F Safaoui MN Helf SC . Student academic performance factors affecting matching into first-choice residency and competitive specialties. BMC Med Educ. 2019;19(1):241. doi:10.1186/s12909-019-1669-9 31262294 PMC6604174

[7] Hasley HL O’Malley GR Jr Bala S Weisman HE Roth PA . Realistic assessment of research publications by neurosurgery residency applicants. World Neurosurg. 2023;172:e372-e377. doi:10.1016/j.wneu.2023.01.030 36646416

[8] McDonald M Khan S Cabatu C Scott F . Osteopathic orthopaedic residency selection criteria: program directors’ survey and analysis. Spartan Med Res J. 2020;4(2):11598. doi:10.51894/001c.11598 33655168 PMC7746051

[9] Beckman JJ Speicher MR . Characteristics of ACGME residency programs that select osteopathic medical graduates. J Grad Med Educ. 2020;12(4):435-440. doi:10.4300/JGME-D-19-00597.1 32879683 PMC7450739

[10] Hansen E Pilarski A Plasner S Cheaito MA Epter M Kazzi A . The osteopathic applicant. J Emerg Med. 2019;56(4):e65-e69. doi:10.1016/j.jemermed.2018.11.003 30979408

[11] Heard MA Buckley SE Burns B Conrad-Schnetz K . Identifying attitudes toward and acceptance of osteopathic graduates in surgical residency programs in the era of single accreditation: results of the American College of Osteopathic Surgeons Medical Student Section questionnaire of program directors. Cureus. 2022;14(3):e22870. doi:10.7759/cureus.22870 PMC8982488 35399472

[12] Stobart-Gallagher M Smith L Giordano J , et al. Recommendations from the council of emergency medicine residency directors: osteopathic applicants. West J Emerg Med. 2019;20(1):111-116. doi:10.5811/westjem.2018.9.39814 30643612 PMC6324709

[13] Tvedten EJ Turnbull JP Guo W Motaparthi K . Attitudes toward allopathic and osteopathic candidates in the dermatologic residency application process. Clin Dermatol. 2023;41(1):178-186. doi:10.1016/j.clindermatol.2022.10.002 36252728

[14] Drake JA Diggs LP Martin SP , et al. Characteristics of matriculants to thoracic surgery residency training programs. Ann Thorac Surg. 2021;112(6):2070-2075. doi:10.1016/j.athoracsur.2020.12.017 33378696 PMC9913615

[15] National Resident Matching Program. Charting Outcomes in the Match: Senior Students of U.S. Osteopathic Medical Schools; 2018 Main Residency Match. National Resident Matching Program; 2018:9-13.

[16] National Resident Matching Program. Charting Outcomes in the Match: Senior Students of U.S. Osteopathic Medical Schools; 2020 Main Residency Match. National Resident Matching Program; 2020:9-14.

[17] National Resident Matching Program. Charting Outcomes in the Match: Senior Students of U.S. Osteopathic Medical Schools; 2022 Main Residency Match. National Resident Matching Program; 2022:9-14.

[18] National Resident Matching Program. Charting Outcomes in the Match: Senior Students of U.S. Osteopathic Medical Schools; 2024 Main Residency Match. National Resident Matching Program; 2024:9-13.

[19] Matthews CN Estrada DC George-Weinstein M Claeson KM Roberts MB . Evaluating the influence of research on match success for osteopathic and allopathic applicants to residency programs. J Am Osteopath Assoc. 2019;119(9):588-596. doi:10.7556/jaoa.2019.102 31449305

[20] Etheart I Krise SM Burns JB Conrad-Schnetz K . The effect of single accreditation on medical student match rates in surgical specialties. Cureus. 2021;13(4):e14301. doi:10.7759/cureus.14301 PMC8099006 33968513

